# Altered lignocellulose chemical structure and molecular assembly in *CINNAMYL ALCOHOL DEHYDROGENASE*-deficient rice

**DOI:** 10.1038/s41598-019-53156-8

**Published:** 2019-11-20

**Authors:** Andri Fadillah Martin, Yuki Tobimatsu, Ryosuke Kusumi, Naoyuki Matsumoto, Takuji Miyamoto, Pui Ying Lam, Masaomi Yamamura, Taichi Koshiba, Masahiro Sakamoto, Toshiaki Umezawa

**Affiliations:** 1 Research Institute for Sustainable Humanosphere, Kyoto University, Uji, Kyoto 611-0011 Japan; 2 Research Center for Biotechnology, Indonesian Institute of Sciences (LIPI), Jl. Bogor KM 46, Cibinong, Bogor 16911 Indonesia; 3 Graduate School of Agriculture, Kyoto University, Sakyo-ku, Kyoto 606-8502 Japan; 4 Research Unit for Development of Global Sustainability, Kyoto University, Uji, Kyoto 611-0011 Japan

**Keywords:** Molecular engineering in plants, Bioalcohols

## Abstract

Lignin is a complex phenylpropanoid polymer deposited in plant cell walls. Lignin has long been recognized as an important limiting factor for the polysaccharide-oriented biomass utilizations. To mitigate lignin-associated biomass recalcitrance, numerous mutants and transgenic plants that produce lignocellulose with reduced lignin contents and/or lignins with altered chemical structures have been produced and characterised. However, it is not fully understood how altered lignin chemistry affects the supramolecular structure of lignocellulose, and consequently, its utilization properties. Herein, we conducted comprehensive chemical and supramolecular structural analyses of lignocellulose produced by a rice *cad2* mutant deficient in *CINNAMYL ALCOHOL DEHYDROGENASE* (*CAD*), which encodes a key enzyme in lignin biosynthesis. By using a solution-state two-dimensional NMR approach and complementary chemical methods, we elucidated the structural details of the altered lignins enriched with unusual hydroxycinnamaldehyde-derived substructures produced by the *cad2* mutant. In parallel, polysaccharide assembly and the molecular mobility of lignocellulose were investigated by solid-state ^13^C MAS NMR, nuclear magnetic relaxation, X-ray diffraction, and Simon’s staining analyses. Possible links between *CAD*-associated lignin modifications (in terms of total content and chemical structures) and changes to the lignocellulose supramolecular structure are discussed in the context of the improved biomass saccharification efficiency of the *cad2* rice mutant.

## Introduction

In the secondary cell walls of vascular plants, lignin and structural polysaccharides, i.e., cellulose and hemicelluloses, are intricately interwoven through both covalent and non-covalent linkages, making up a complex lignocellulose biocomposite^1^. Lignin, a phenylpropanoid polymer accounting for 15%–30% of lignocellulose, has long been recognized as a key recalcitrant factor limiting the efficiency of lignocellulose deconstruction and downstream processing in polysaccharide-oriented biomass utilization processes, for example, those used in the production of pulp and paper and the generation of fermentable sugars for biofuels and biomaterials^2^. To mitigate lignin-associated biomass recalcitrance, numerous transgenic plants that produce lignocellulose with lower lignin contents and/or lignins with altered chemical structures have been generated via up- and/or down-regulation of genes in lignin biosynthesis. Indeed, some lignin-modified mutant and transgenic plants have improved biomass characteristics. For example, reducing the lignin content is a common strategy to improve the extractability and the subsequent enzymatic saccharification of cell wall polysaccharides into fermentable sugars^3–9^. Changing lignin structure, e.g., by altering the composition of aromatic units, distributions of inter-monomeric linkages, and end-unit types in lignin polymers, can also improve the processability of polysaccharide components^6–9^.

As such, there is an increasing body of evidence showing that changes in lignin contents and/or structure affect the supramolecular structure of lignocellulose, i.e., the organization, assembly, and interactions among lignin and polysaccharides in cell walls, and such changes in lignocellulose supramolecular structure ultimately affect the biomass utilization properties. However, largely because of the technical challenges in characterising the highly complex and heterogeneous structure of lignocellulose, only a few studies have attempted to analyse the supramolecular structure of lignocellulose in lignin-related mutants and transgenic plants^10–12^. Consequently, there is still much to learn about how changes in lignin chemistry affect the supramolecular structure of lignocellulose and its utilization properties.

**Figure 1:**
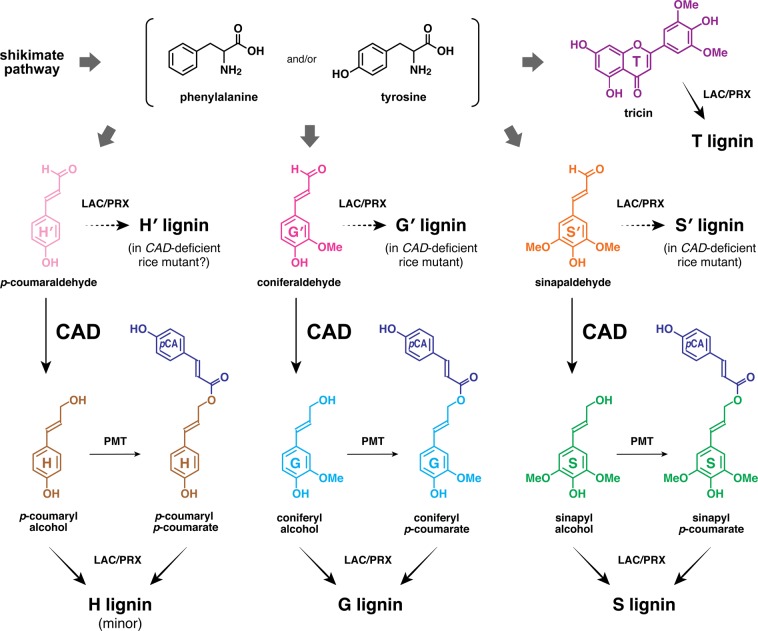
Major lignin monomers and their biosynthesis in grasses. Cinnamyl alcohol dehydrogenase (CAD) catalyses the reduction of hydroxycinnamaldehydes derived from phenylalanine and/or tyrosine into monolignols (hydroxycinnamyl alcohols). PMT, *p*-coumaroyl-CoA:monolignol transferase. LAC, laccase; PRX, peroxidase. H, *p*-hydroxyphenyl. G, guaiacyl. S, syringyl. *p*CA, *p*-coumarate. T, tricin. H′, *p*-coumaraldehyde-derived *p*-hydroxyphenyl. G′, coniferaldehyde-derived guaiacyl. S′, sinapaldehyde-derived syringyl.

In this study, we conducted in-depth structural analyses of the altered lignocellulose produced by a grass mutant deficient in *CINNAMYL ALCOHOL DEHYDROGENASE* (*CAD*), a key enzyme gene involved in lignin biosynthesis. CAD is responsible in the final stage of the biosynthesis of lignin monomers, reducing hydroxycinnamaldehyde precursors into their corresponding hydroxycinnamyl alcohols, i.e., monolignols (Fig. 1)^13^. The resultant monolignols are then exported to cell walls and polymerised into lignins through oxidative radical coupling mediated by wall-localized laccases and peroxidases^14^. In general, monolignols are the only lignin monomers in gymnosperms and eudicots, whereas, in monocotyledonous grasses (Poaceae) including rice tested in this study, a significant proportion of monolignols are γ-acylated with *p*-coumarate before they undergo co-polymerization with non-acylated monolignols (Fig. 1)^15^. In addition, grass lignins also incorporate a flavone, tricin, as a canonical lignin monomer. Tricin can be also co-polymerized with non-acylated and γ-acylated monolignols via radical coupling upon cell wall lignification in grasses (Fig. 1)^16^.

Loss of function or down-regulation of CAD typically leads to unusual incorporation of hydroxycinnamaldehydes into lignins, often accompanied by reddish brown colouration of xylem tissues^17,18^. In grasses, such as maize^19–21^, *Sorghum*^22,23^, rice^24–26^ and *Brachypodium*^27^, mutations in *CAD* genes are associated with the *brown midrib* phenotype that often shows enhanced forage digestibility and/or biomass saccharification efficiency. Likewise, *CAD*-deficient transgenic plants, including non-grass species, often show improved forage digestibity^28^ and better properties for various biomass utilization processes such as chemical pulping^29–31^ and/or enzymatic saccharification^21,24,32–35^. In addition, *CAD*-deficient mutant and transgenic plants may display altered cell wall mechanical properties^25,36–39^. Thus, *CAD*-associated lignin modifications, e.g., reduced lignin contents and/or structural changes upon incorporation of hydroxycinnamaldehydes, may lead to substantial changes in the supramolecular structure of lignocellulose, ultimately leading to improved biomass processabity and altered cell wall mechanical properties^11,12^. However, our understanding of this phenomenon at the molecular level remains limited. It is therefore important to investigate the relationship between changes in chemical structure and the supramolecular structure of lignocellulose produced by *CAD*-deficient plants.

Herein, we conducted comprehensive chemical and supramolecular structural analyses of lignocellulose produced by a *CAD*-deficient mutant of rice (*Oryza sativa* L. spp. *japonica* cv. Nipponbare). Previously, we reported that the *cad2* (*cinnamyl alcohol dehydrogenase 2*) rice mutant, which harbours a *Tos17* retrotransposon insertion in the second exon of *OsCAD2* (Os02g0187800), displays a typical *brown midrib* phenotype and enhanced biomass saccharification efficiency along with altered lignin content and structure as determined for different rice plant tissues including internode culms examined in this study primarily by conventional wet-chemical methods (e.g., thioglycolic acid lignin assay, nitrobenzene oxidation and thioacidolysis)^24^. In the present study, to further dissect the altered lignin structures in the *cad2* rice mutant, we used solution-state two-dimensional (2D) ^1^H–^13^C heteronuclear single-quantum coherence (HSQC) NMR and complementary chemical methods; although a number of studies applied modern HSQC approaches to analyse altered lignins produced by *CAD*-deficient eudicot plans^34,35,40–44^, no study to date has used such techniques for analysis of *CAD*-deficient grass lignins. In addition, for the first time for a lignin-associated *CAD* mutant, we performed an integrative analysis of lignocellulose assembly and molecular mobility in the *cad2* rice cell wall using solid-state ^13^C magic-angle-spinning (MAS) NMR spectroscopy, nuclear magnetic relaxation, wide-angle X-ray diffraction (WAXD), and Simon’s staining analyses. The results of this study provide a molecular basis for understanding the relationships among *CAD*-associated lignin structural modifications, changes in the lignocellulose supramolecular structure, and biomass saccharification efficiency.

## Results

The *cad2* rice mutant^24^ was re-cultivated side-by-side with wild-type (control) rice. As previously demonstrated^24^, the *cad2* mutant displayed typical *brown midrib* and *gold hull and internode* phenotypes, exhibiting reddish-brown colouration of the midrib, grains (hulls), and culm straws (Fig. S1). Under controlled greenhouse conditions, the *cad2* mutant displayed overall similar growth performance to that of wild-type rice in terms of plant height, culm length, tillering, and biomass production (Table S1). Also, as previously reported^24^, the extractive-free cell wall residue (CWR) samples prepared from mature culm tissues of the *cad2* mutant displayed significantly enhanced enzymatic saccharification efficiency over that of the wild type (Table S2). The enhancement of saccharification efficiency after 24 h of incubation with a cocktail of cellulolytic enzymes^45^ was ca. 32% when expressed as glucose yield per unit cell walls and ca. 36% when expressed as glucose yield per unit total glucan (Table S2), similar to the data reported previously^24^. The rice culm CWR samples were then subjected to in-depth cell wall chemical and supramolecular structure analyses.

### Chemical analyses of *CAD*-deficient rice cell walls

**Figure 2:**
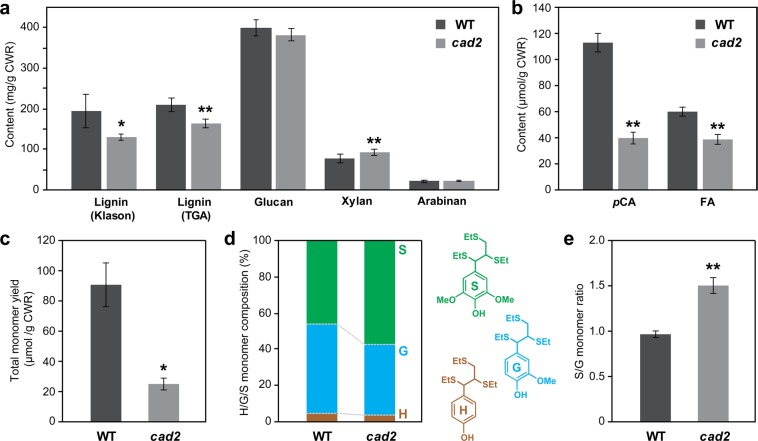
Chemical analysis data of culm cell walls from wild-type (WT) and *cad2* mutant rice plants. (**a**) Lignin content determined by Klason and thioglycolic acid (TGA) assays and sugar content determined by neutral sugar analysis. (**b**) Alkaline-releasable p-coumaric acid (*p*CA) and ferulic acid (FA) contents. (**c**–**e**) Lignin composition analysis by thioacidolysis. Total yield (**c**); composition (**d**); and S/G molar ratio (**e**) of H, G, and S-type trithioethylpropane monomers released from H, G, and S-type β–O–4 lignin units. Values are means ± standard deviation (SD) from individually analysed plants (*n* = 3). Asterisks indicate significant differences between WT and *cad2* mutant plants (Student’s *t*-test, *p < 0.05; **p < 0.01). CWR, cell wall residue.

Consistent with previously reported data^24^, the lignin content in the *cad2* mutant culm cell walls was significantly reduced, by ca. 30% and 20% as determined by the Klason and thioglycolic acid lignin assays, respectively, compared with that in wild-type culm cell walls (Fig. 2a). The xylan content was significantly increased by ca. 21% in the *cad2* mutant cell walls compared with that in the wild-type cell walls, whereas the contents of crystalline and amorphous glucans and arabinans in cell walls did not differ significantly between *cad2* and wild type (Fig. 2a). These results suggest that the increase in xylan content at least partially compensates for the reduced lignin content in *cad2* cell walls. Cell wall-bound *p*-coumarates (*p*CA) and ferulates (FA) were quantified as the corresponding free acids released by mild alkaline hydrolysis of CWRs. The *cad2* mutant cell walls displayed largely reduced *p*CA (ca. 65% less compared to wild-type levels) and also mildly reduced FA (ca. 37% less) levels (Fig. 2b). Given that a majority of *p*CA is bound to lignins in typical grass cell walls^15^, the reduced *p*CA levels in the *cad2* mutant cell walls were likely associated with the reduced lignin levels (Fig. 2a). Meanwhile, it is currently unclear why FA, which is mostly bound to arabinose residues in grass hemicelluloses rather than to lignins^46^, was reduced in the *cad2* mutant cell walls even with no significant change in arabinose content (Fig. 2a).

Analytical thioacidolysis quantifies lignin-derived monomeric compounds released by the chemical cleavage of the major β–O–4 linkages in lignin polymers^47^. The *cad2* mutant cell walls released considerably less monomeric compounds (ca. 73% less) upon the thioacidolysis degradation than did the cell walls of wild type (Fig. 2c). This could be attributed not only to the reduced lignin content (Fig. 2a) but also to the replacement of the normal β–O–4 linkages by unusual hydroxycinnamaldehyde-derived linkages in lignin polymers. In fact, we previously demonstrated that thioacidolysis of the internode cell wall samples prepared from the *cad2* mutant lines released indene derivatives^24^ which have been used as useful molecular markers for atypical hydroxycinnamaldehyde-derived 8–O–4 linkages generated upon *CAD*-deficiency^48^. In addition, the proportion of guaiacyl (G)-type lignin monomers out of total lignin monomers [G, syringyl (S), and *p*-hydroxyphenyl (H)-type monomers] was considerably depleted in the *cad2* mutant cell walls (Fig. 2d). Consequently, the S/G ratio in cell walls was considerably higher for the *cad2* mutant than for wild type (Fig. 2e). These results suggest that the *OsCAD2*-knockout mutation preferentially affects G-type lignin polymer units in rice, as further examined by 2D NMR below.

### 2D HSQC NMR analysis of altered lignin structures in *CAD*-deficient rice cell walls

**Figure 3:**
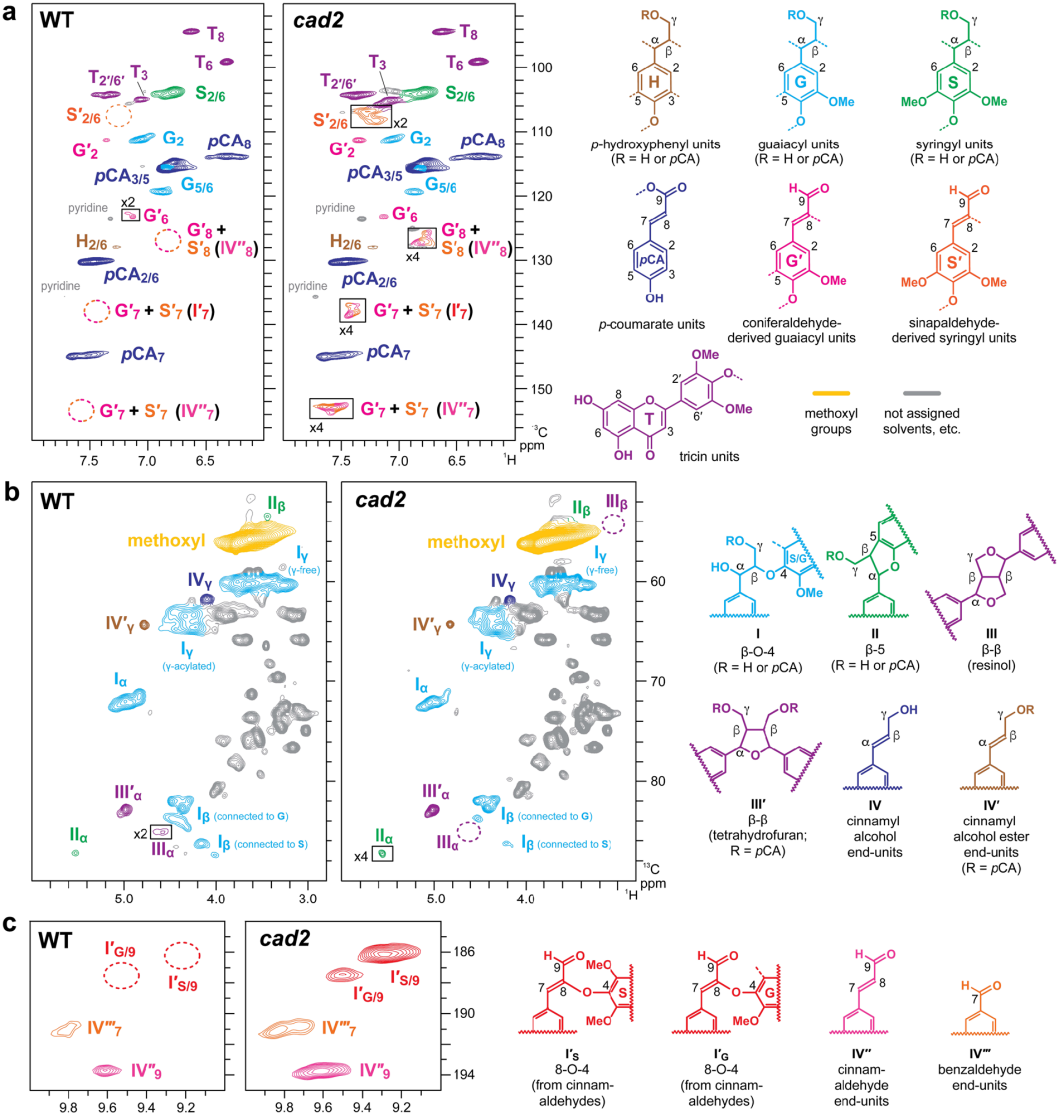
Solution-state two-dimensional short range ^1^H–^13^C correlation (HSQC) NMR analysis of dioxane-soluble lignin samples prepared from mature culm tissues of wild-type (WT) and *cad2* mutant rice plants. (**a**) Aromatic sub-regions showing signals from major lignin aromatic units. (**b**) Oxygenated aliphatic sub-regions showing signals from major lignin side-chain units. (**c**) Aldehyde sub-regions showing signals from aldehyde units. Contours are color-coded to match the displayed structures. Boxes labelled ×2 and ×4 represent regions with scale vertically enlarged by 2- and 4-folds, respectively. Volume integration data are listed in Table 1. For signal assignments, see Tables S3 and S4 in Supplementary Information.

To further investigate changes in the chemical structure of lignin in the *cad2* mutant rice, we performed 2D HSQC NMR analysis^49,50^ on lignin-enriched cell wall samples which were prepared via enzymatic removal of cell wall polysaccharides from CWRs^50,51^ and also on dioxane-soluble lignin samples which were extracted from the lignin-enriched cell walls with a dioxane-water solvent for further lignin purification^51^. The aromatic sub-regions of the HSQC spectra of the wild-type lignin-enriched cell wall (Fig. S2a) and dioxane-soluble lignin (Fig. 3a) samples displayed typical lignin aromatic signals from G, S and H units (**G**, **S**, and **H**, respectively) along with the signals from *p*-coumarate (***p*CA**) and tricin (**T**) units (Table S3). These results are highly consistent with the fact that wild-type rice lignins are mostly derived from polymerization of γ-free and γ-*p*-coumaroylated monolignols and tricin^52,53^, as is typical for most grass lignins (Fig. 1)^9^.

The proportions of hydroxycinnamaldehyde-derived G-type (**G′**) and S-type (**S′**) lignin units (Table S3)^34,35,40–44^ in wild-type lignins were estimated to be 6.3% and 3.2% per total monolignol-derived (**G** + **S** + **H**) and hydroxycinnamaldehyde-derived (**G′** + **S′**) aromatic units in lignin-enriched cell wall and dioxane-soluble lignin spectra, respectively, based on the volume integration analysis of the C2–H2 contour intensities (Table 1). It should be noted that the **G′**_**2**_ and **S′**_**2/6**_ signals could be overestimates of such hydroxycinnamaldehyde-derived lignins, especially in lignin-enriched cell wall spectra, as these signals can overlap with those from ferulates in residual arabinoxylans^52,54^ and also oxidized β-aryl ether lignin units with α-carbonyl carbons^44^. Both the **G′** and **S′** signals were clearly augmented in the *cad2* spectra (Figs. 3a and S2a, Table 1). The **G′** signals were more prominently augmented (to ca. 19% and 16% in lignin-enriched cell wall and dioxane-soluble lignin spectra, respectively) than **S′** signals (to ca. 2% and 3% in lignin-enriched cell wall and dioxane-soluble lignin spectra, respectively) in all the HSQC spectra collected for *cad2* mutant samples (Table 1). Consequently, consistent with the above thioacidolysis-derived compositional data (Fig. 2e), the **S**/**G** ratio was significantly increased in *cad2* (by ca. 70% and 45% in lignin-enriched cell wall and dioxane-soluble lignin spectra, respectively), whereas the overall ratio of S-type and G-type lignin aromatic signals, i.e., (**S** + **S′**)/(**G** + **G′**), was similar between *cad2* and wild-type spectra (Table 1). In addition, the conjugated C7–C8 double-bond signals from hydroxycinnamaldehyde-derived 8–O–4-units (**I′**) and cinnamaldehyde (**IV′**′) end-units (Table S3)^34,35,40–44^ were clearly visible in the *cad2* spectra, but were practically absent in the wild-type spectra (Figs. 3a and S2a), supporting our notion that hydroxycinnamaldehyde-derived lignins are considerably augmented in the *cad2* mutant cell walls.

**Table 1:** Volume integration data of major lignin signals in HSQC spectra of wild-type (WT) and *cad2* mutant culm cell wall and lignin samples.

	Lignin-enriched cell walls^a^		Dioxane-soluble lignins^b^	
	WT	*cad2*	WT	*cad2*
**Aromatic units**				
**H** (*p*-Hydroxyphenyl units) (%)^c^	5.0 ± 1.3	6.6 ± 0.9	2.0	2.9
**G** (Guaiacyl units) (%)^c^	45.1 ± 3.3	27.1 ± 3.0**	49.4	30.7
**S** (Syringyl units) (%)^c^	43.9 ± 2.5	45.0 ± 2.3	44.7	47.9
**S′** (S units from sinapaldehyde) (%)^c^	n.d.	2.0 ± 0.8*	n.d.	2.5
**G′** (G units from coniferaldehyde) (%)^c^	6.3 ± 1.6	19.2 ± 1.0**	3.2	16.1
**S**/**G**	1.0 ± 0.1	1.7 ± 0.3*	1.1	1.6
(**S + S′**)/(**G + G′**)	0.9 ± 0.1	1.0 ± 0.1	1.0	1.1
**T** (Tricin units) (%)^c^	14.3 ± 2.0	18.8 ± 2.4	24.1	43.3
***p*CA** (*p*-Coumarate units) (%)^c^	89.7 ± 9.6	100.6 ± 12.5	91.6	102.4
**Inter-monomeric and end-group units**				
**I** (β–O–4) (%)^d^	85.3 ± 1.0	81.1 ± 1.6*	82.7	80.6
**II** (β–5) (%)^d^	2.3 ± 0.5	trace**	5.5	0.9
**III** + **III′** (β–β) (%)^d^	12.4 ± 0.7	18.7 ± 1.2**	11.8	18.6
**I′** (8–O–4 from cinnamaldehydes) (%)^d^	n.d.	7.9 ± 1.4*	n.d.	18.9
**IV′′** (cinnamaldehyde end-units) (%) ^d^	trace	6.4 ± 2.6*	0.8	13.5
**IV′′′** (benzaldehyde end-units) (%)^d^	trace	2.0 ± 0.2**	trace	2.2

^a^Values are means ± standard deviation (SD) for three individually analyzed plants. Asterisks indicate significant differences between WT and *cad2* mutant plants (Student’s *t*-test, *p < 0.05; **p < 0.01). ^b^Values are data collected for culm cell samples pooled from three biological replicates for each line. ^c^Expressed as a percentage of the total of **H**, **G**, **S**, **G′** and **S′** aromatic signals. ^d^Expressed as a percentage of the total of **I**, **II**, **III**, and **III′** inter-monomeric linkage signals. For structure abbreviations and peak assignments, also see Fig. 3 and Tables S3 and S4. CWR, cell wall residue. n.d., not detected.

The proportions of **H** signals were relatively small (less than 7%) and similar between *cad2* and wild-type spectra (Table 1). The abundance of ***p*CA** and **T** signals was also not significantly different between *cad2* and wild-type spectra. Given that *p*-coumarates are mostly attached to S units in the cell walls of rice^54,55^ and other typical grasses^15^, that the proportions of *p*-coumarate units were similar in *cad2* and wild-type cell walls is in line with the observation that the proportions of S units were also similar between them (Table 1). Taken together, these results show that the *OsCAD2*-knockout mutation promotes the incorporation of non-canonical coniferaldehyde instead of canonical coniferyl alcohol into lignin, with much less effect on the incorporation of non-canonical sinapaldehyde instead of canonical sinapyl alcohol and sinapyl *p*-coumarate, and no apparently significant effect on the incorporation of tricin into lignin (Fig. 1).

**Figure 4:**
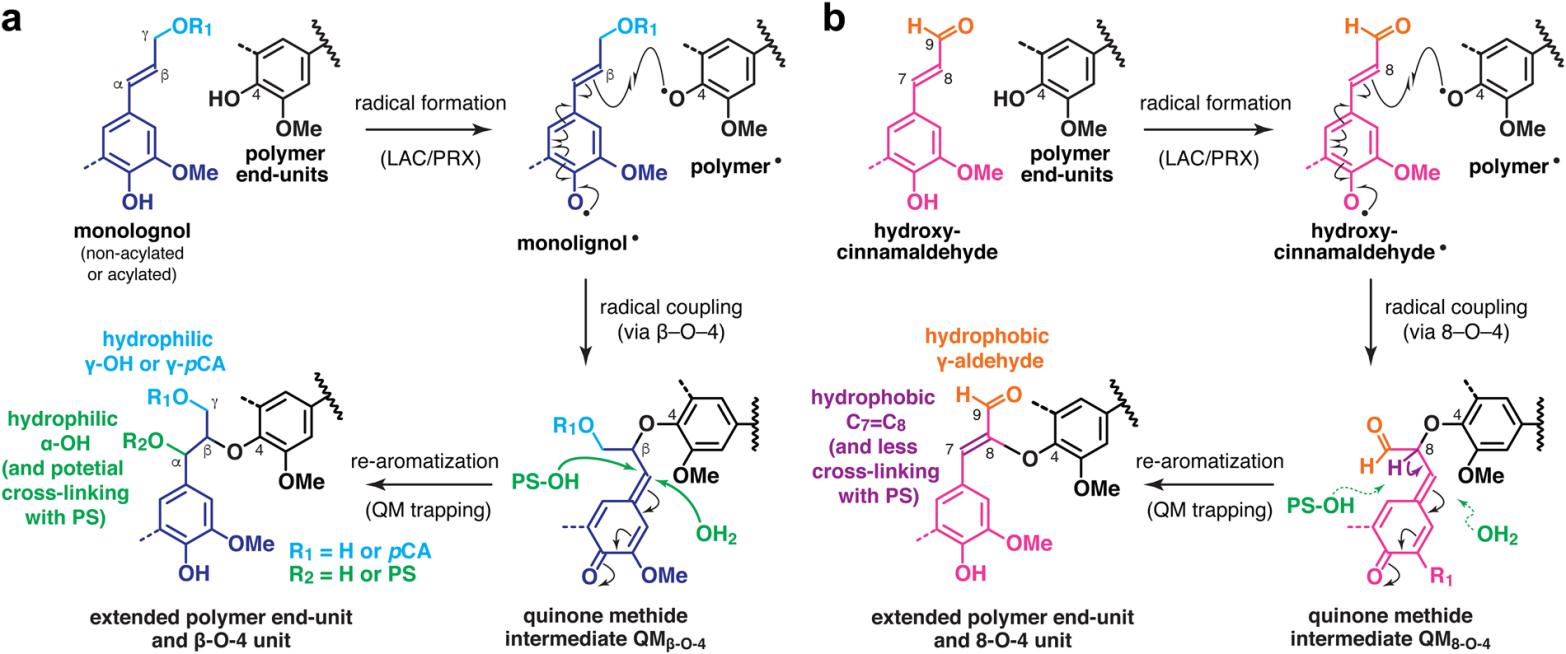
Cross-coupling reactions of canonical non-acylated and acylated monolignols (**a**) and non-canonical hydroxycinnamaldehydes (**b**) giving rise to the β–O–4 and 8–O–4 linkage units, respectively, in lignin polymers. *p*CA, *p*-coumarate. PS, polysaccharides. QM, quinone methide intermediates. LAC, laccase; PRX, peroxidase.

The oxygenated-aliphatic (Figs. 3b and S2b) and aldehyde (Figs. 3c and S2c) sub-regions of the HSQC spectra show the distributions of the various inter-monomeric and end-unit linkage types in the lignin polymers (Table S4). In line with the above aromatic compositional analysis data, the aldehyde C9–H9 signals from hydroxycinnamaldehyde-derived 8–O–4-units (**I′**) as well as those from cinnamaldehyde (**IV′′**) and benzaldehyde (**IV′′′**) end-units^34,35,40–44^ were clearly visible in the *cad2* spectra, but were much smaller or practically absent in the wild-type spectra (Figs. 3c and S2c). Collectively, the appearance of the atypical 8–O–4-units (**I′**) bearing 9-aldehyde and unsaturated C7–C8 double bonds provided evidence for the participation of hydroxycinnamaldehydes in oxidative radical coupling during lignification (Fig. 4)^40,42^. Volume integration analysis determined that **I′** signals account for ca. 8% and 19% of the total of major lignin linkage signals (**I + II + III** + **III**′) in the *cad2* lignin-enriched cell wall and dioxane-soluble lignin spectra, respectively (Table 1). Meanwhile, the relative signal intensities of the conventional β–O–4 (**I**) and β–5 (**II**) units were proportionally decreased and those of β–β linkages (**III** + **III′**) were increased in the *cad2* spectra compared with those in the wild-type spectra (Table 1). Such decreases and increases in β–5 (**II**) and β–β (**III** + **III′**) units, respectively, are typical consequences of the increased **S**/**G** ratio^34,55,56^. The decrease in β–*O*–4 (**I**) was likely due to its partial replacement by hydroxycinnamaldehyde-derived 8–O–4-units (**I′**)^34,44^.

### Solid-state NMR and WAXD analyses of lignocellulose supramolecular structure in *CAD*-deficient rice cell wall samples

**Figure 5:**
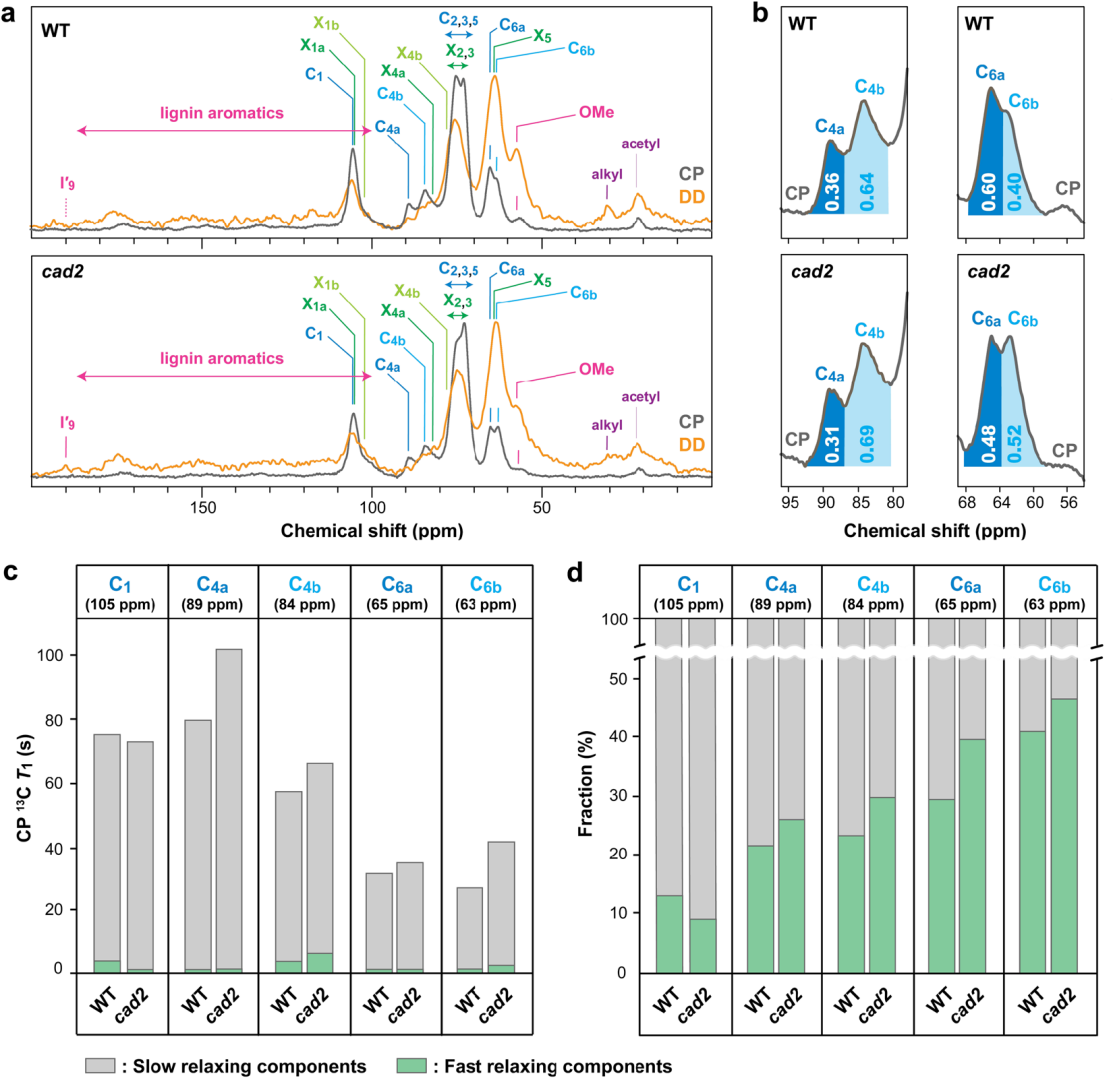
Solid-state ^13^C MAS NMR analyses of culm cell walls from wild-type (WT) and *cad2* mutant rice plants. (**a**) CP and DD MAS NMR spectra collected with 2-second recycling delay. (**b**) Expanded CP MAS spectra showing cellulose C4 and C6 signals. Inserted values are volume integrals for indicated peak areas. (**c**) CP ^13^C *T*_1_ relaxation times for slow- and fast-relaxing components in major cellulose carbon sites. (**d**) Fractions of slow- and fast-relaxing components in major cellulose carbon sites. For signal assignments, see Table 2.

To investigate how the *CAD*-associated lignin modifications affect the supramolecular structure of lignocellulose, we performed an integrated analysis of polysaccharide molecular assembly and mobilities based on solid-state ^13^C MAS NMR spectra, spin-lattice relaxation times (*T*_1_), and WAXD patterns of *cad2* and wild-type culm CWR powder samples. The ^13^C MAS NMR spectra were collected either by using typical ^1^H–^13^C cross-polarization (CP) to detect rigid cellulose components or by ^13^C-directed dipolar decoupling (DD or direct polarization, DP) with a fast 2-s recycling delay to emphasize more mobile components^57–59^, i.e., lignin and hemicelluloses (Fig. 5a). As expected, the CP MAS spectra of both *cad2* and wild-type cell walls were dominated by signals assigned to rigid cellulose backbones with peaks at 105 ppm (**C**_**1**_), 89 and 84 ppm (**C**_**4**_), 75 and 73 ppm (**C**_**2**,**3**,**5**_), and 65 and 63 ppm (**C**_**6**_) (Fig. 5a, Table 2). The DD MAS spectra were apparently enriched with signals from mobile hemicelluloses, mainly xylans, with obvious peaks at 105 ppm (**X**_**1**_), 82 ppm (**X**_**4**_), 76 ppm (**X**_**2**,**3**_), and 64 ppm (**X**_**5**_), as well as signals from mobile lignin aromatic (110–180 ppm) and methoxyl (57 ppm) carbons (Fig. 5a, Table 2)^58,60,61^. In addition, small signals assignable to aldehyde carbonyl carbons in the hydroxycinnamaldehyde-derived lignin units were present, albeit at low intensity, only in the DD spectrum of *cad2* cell walls (Fig. 5a, Table 2).

**Table 2:** Major signal assignments in solid-state ^13^C MAS NMR spectra of rice cell walls.

Assignment	Resonance (ppm)
**I′**_**9**_ (aldehyde lignin C9)	190
**C**_**1**_ (cellulose C1)	105
**X**_**1a**_ (rigid/twofold xylan C1)	105^a^
**X**_**1b**_ (mobile/threefold xylan C1)	103^a^
**C**_**4a**_ (crystalline/internal cellulose C4)	89
**C**_**4b**_ (amorphous/surface cellulose C4)	84
**X**_**4a**_ (rigid/twofold xylan C4)	82^a^
**X**_**4b**_ (threefold/mobile xylan C4)	77^a^
**X**_**2,3**_ (xylan C2 and C3)	75-72^a^
**C**_**2,3,5**_ (cellulose C2, C3 and C5)	75-72
**C**_**6a**_ (crystalline/internal cellulose C6)	65
**X**_**5**_ (xylan C5)	64^a^
**C**_**6b**_ (amorphous/surface cellulose C6)	63
**OMe** (lignin methoxyl CH_3_)	57

^a^Not well resolved in this study. Chemical shift values are according to Simmons *et al*.^60^.

The CP MAS spectra showed glucose C4 and C6 signals from two distinct cellulose environments, i.e., **C**_**4a**_ (89 ppm) and **C**_**6a**_ (65 ppm), which are often assigned to glucose residues in crystalline and/or internal cellulose, and **C**_**4b**_ (84 ppm) and **C**_**6b**_ (63 ppm), which are often assigned to glucose residues in amorphous and/or surface cellulose (Table 2)^57–64^. Interestingly, in our volume integration analysis, the relative signal intensities of the crystalline and/or internal cellulose carbons over the amorphous and/or surface cellulose carbons, i.e., **C**_**4a**_/**C**_**4b**_ and **C**_**6a**_/**C**_**6b**_, were notably decreased in *cad2* cell walls compared with those in wild-type cell walls (Fig. 5b), suggesting that cellulose molecular assembly is compromised in *cad2* cell walls.

**Figure 6:**
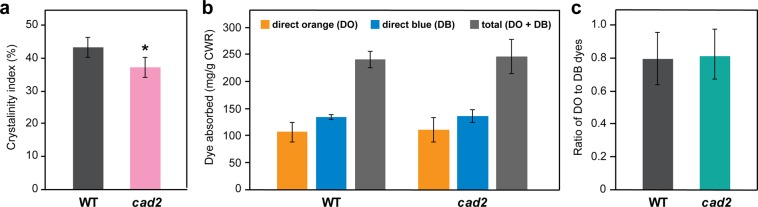
WAXD and Simon’s staining assay of culm cell walls of wild-type (WT) and *cad2* mutant rice plants. WAXD-derived apparent crystallinity index (**a**); amounts of absorbed direct orange (DO) and direct blue (DB) dyes (**b**); and ratio of absorbed DO to DB dyes (**c**) after Simon’s staining. Values are means ± standard deviation (SD) from individually analysed plants (*n* = 3). Asterisks indicate significant differences between WT and *cad2* mutant plants (Student’s *t*-test, *p < 0.05). CWR, cell wall residue.

To quantitatively assess the defective cellulose assembly, we collected WAXD profiles of *cad2* and wild-type cell walls (Fig. S3) and determined their apparent crystallinity indices as expressed by the intensity ratio of the crystalline and amorphous scatters (Fig. 6a)^65^. In line with our assessment by CP MAS NMR (Fig. 5b), the crystallinity index of cell walls was significantly decreased, by ca. 15%, in the *cad2* mutant compared with wild type (Fig. 6a). Taken together, these data suggest that *cad2* cell walls have less well-defined cellulose assembly compared with that in wild-type cell walls.

Next, we measured site-specific ^13^C spin-lattice relaxation times (*T*_1_) to investigate the mobility of cellulose in *cad2* and wild-type cell walls. Because we used the common Torchia pulse sequence^66^, which uses CP for the initial ^13^C magnetization, the collected relaxation data reflect the molecular motion of mainly CP-enhanced rigid cellulose components. The relaxation data collected for major cellulose carbon sites exhibited double exponential decays with distinctively slow- (*T*_1_ > 20 s) and fast- (*T*_1_ < 5 s) relaxing components, each representing relatively rigid and mobile domains, respectively, both in the crystalline/internal and amorphous/surface cellulose backbones. This pattern was similar to the Torchia-CP relaxation profiles that have been reported for powdery biomass and cellulose samples in other studies^62–64,67^. Intriguingly, the *cad2* cell wall samples tended to display higher *T*_1_ values than those of wild-type cell wall samples at most of the cellulose carbon sites, except for **C**_**1**_ (105 ppm) (Fig. 5c). However, most of the cellulose carbon sites, again except for **C**_**1**_, in *cad2* cell wall samples showed higher fractional weighing for faster-relaxing components over slower-relaxing components compared with those in wild-type cell wall samples (Fig. 5d). The exceptional behaviour of *T*_1_ and the fractional weighing of faster and slower components at the **C**_**1**_ site might be due to the contributions of overlapping xylan **X**_**1**_ (Table 2) as well as syringyl (**S**_**2/6**_) and tricin (**T**_**2′/6′**_) lignin aromatic carbons (Fig. 3a; Table S3) at this particular site (δ_C_ = ca. 105 ppm). Overall, these relaxation data suggest that not only the assembly but also the molecular mobility of cellulose molecules are somehow affected in *cad2* rice cell walls.

### Simon’s staining assay to assess surface accessibility of cellulose in *CAD*-deficient rice cell wall samples

Lastly, we used Simon’s staining assay^68,69^ to test whether or not the enhanced saccharification performance of *cad2* mutant cell walls (Table S2)^24^ is associated with the physical accessibility of cellulolytic enzymes to the cellulose substrate. This assay semi-quantitatively determines the amplitudes of the pore surface area and accessibility of cellulose substrates in biomass by applying two cellulose binding dyes, i.e., direct orange (DO) and direct blue (DB), with distinct colours, molecular sizes, and cellulose binding affinities; in particular, DO resembles the molecular size of typical cellulases with a high affinity for cellulose^68,69^. In the assay, we observed no significant differences between *cad2* and wild-type control cell wall powders in terms of cellulose porosity and accessible surface area, as assessed by the amount of the adsorbed dyes (Fig. 6b) or by the ratio of the adsorbed large DO to small DB dyes (Fig. 6c)^68,69^. These results indicate that, at least within the limitation of the capability of Simon’s staining assay, the porosity and physical accessability of cellulose are not significantly compromised in *cad2* rice cell walls.

## Discussion

### *OsCAD2*-deficient rice produces aldehyde-enriched lignins mainly from polymerization of coniferaldehyde

As envisioned, our HSQC NMR analysis revealed that cell walls produced in *cad2* rice mutants are augmented in unusual aldehyde-enriched lignins. An estimation based on the HSQC contour intensities suggested that ca. 19–21% of the mutant lignins are from the polymerization of the atypical hydroxycinnamaldehydes replacing the conventional non-acylated and acylated monolignols (Table 1). The results of both the HSQC NMR and thioacidolysis analyses suggest that the *OsCAD2*-knockout mutation results in the replacement of mainly the G-type lignin monomer, i.e., coniferyl alcohol (and coniferyl *p*-coumarates, albeit with low levels), with the corresponding G-type hydroxycinnamaldehyde, i.e., coniferaldehyde, with much less impact on the replacement of the S-type lignin monomers, i.e., sinapyl alcohol and sinapyl *p*-coumarate (Fig. 1). The HSQC NMR analysis estimated that ca. 87–91% of the hydroxycinnamaldehyde-derived lignins in *cad2* rice cell walls are from polymerization of coniferaldehyde, with the rest from sinapaldehyde (Table 1). Meanwhile, it was apparent that the null mutation of *OsCAD2* in our *cad2* mutant^24^ does not completely eliminate total CAD activity for lignification, because HSQC NMR signals from the typical lignin polymer units are still prominent in the spectra of *cad2* mutant cell walls (Fig. 2, Table 1); in contrast, the *Arabidopsis cadc cadd* double mutant^34^ and *Medicago truncatula cad1* mutant^44^ produce lignins largely dominated (>95% based on 2D HSQC NMR) by unusual lignin units derived from polymerization of hydroxycinnamaldehydes, and almost completely devoid of normal lignin units derived from polymerization of canonical monolignols. Given that the rice genome encodes at least 11 CAD and CAD-like proteins besides OsCAD2^70,71^, it is conceivable that at least some of them play a role along with OsCAD2 in rice lignification, and may particularly function in the biosynthesis of S-type lignins. Further genetic and biochemical characterizations of rice CAD and CAD-like proteins are needed to explore their specific functions.

### Lignocellulose assembly is compromised in *CAD*-deficient rice cell walls

The results of the solid-state ^13^C MAS NMR, spin-lattice relaxation, and WAXD analyses of rice cell wall powder samples indicated that cellulose assembly and mobility are disrupted in the *CAD*-deficient rice mutant. In particular, *cad2* mutant cell wall samples have less well-defined cellulose assembly compared with that in the wild type, as assessed based on the decreased peak areas of the crystalline/internal carbons over the amorphous/surface carbons of cellulose in the ^13^C CP MAS spectra (Fig. 5b) as well as the decreased ratio of crystalline to amorphous scatters in the WAXD profiles (Fig. 6a). Concomitantly, the site-specific spin-lattice relaxation (*T*_1_) times were increased for most of the cellulose carbons in both the crystalline/internal and the amorphous/surface cellulose backbones (Fig. 5c), whereas the fractional weighing of faster-relaxing (more mobile) components was increased over that of slower-relaxing (more rigid) components at most of the cellulose carbon sites in the *cad2* mutant cell wall samples (Fig. 5d), suggesting complex changes in cellulose mobility.

Overall, our data are in line with those reported by Liu *et al*.^12^ who also detected disordered orientations of cellulose fibrils in inflorescence stems of *CAD*-deficient *Arabidopsis cadc cadd* mutant lines using high-resolution scanning X-ray microdiffraction^12^. A reduced lignin content (Fig. 2a) may represent the major factors causing such disintegration of lignocellulose in the *cad2* mutant rice tested in this study. In fact, Ruel et al. observed altered organization of cellulose microfibrils in a lignin-reduced Arabidopsis *ccr1* mutant deficient in *CINNAMOYL-CoA REDUCTASE* (*CCR*)^10^. Notably, however, not only reduced lignin content but also modified lignin chemical structures, i.e., incorporation of hydroxycinnamaldehydes, upon *CAD*-deficiency can affect cellulose molecular assembly, albeit perhaps with less impacts in our rice *cad2* mutant containing lignin with ca. 20% (based on 2D HSQC NMR, Table 1) of the hydroxycinnamaldehyde-derived units compared to those in the previously reported *CAD*-deficient mutant plants with lignins much more dominated (>95% based on 2D HSQC NMR) by the hydroxycinnamaldehyde-derived units^34,44^.

Our current models of lignocellulose supramolecular structure envision that, whereas direct associations between lignin and cellulose are unlikely, hemicelluloses, e.g., xylans and mannans, integrate lignin and cellulose by forming non-covalent and/or covalent linkages independently with the two, although it is yet difficult to test these models experimentally^60,61,72–74^. It is therefore plausible that the disordered cellulose assembly observed in the cell walls of *CAD*-deficient rice (this study) and *Arabidopsis*^12^ result from the disruption of lignin–hemicellulose associations upon lignin modifications associated with *CAD*-deficency. For example, the incorporation of hydroxycinnamaldehydes may increase the hydrophobicity of lignin polymers. As further demonstrated in this study, hydroxycinnamaldehydes produce the 9-aldehyde groups that replace the typical γ-alcohol or γ-*p*-coumarate groups, as well as the unusual 8–O–4 linkages with unsaturated C7–C8 double bonds that replace the typical β–O–4 linkages with α-hydroxyl groups in lignin polymers (Fig. 4)^40,42^. A previous study using molecular dynamic modelling suggested that such *CAD*-associated structural modifications of lignin polymers that make the polymers more hydrophobic may reduce non-covalent lignin–hemicellulose interactions in cell walls^11^. In addition, there may be fewer covalent linkages between lignin and hemicelluloses in *CAD*-deficient cell walls. In the course of typical lignin polymerization from the conventional non-acylated and acylated monolignols, the quinonemethide intermediate resulting from β–O–4 cross-coupling can be trapped for re-aromatization by a hydroxyl or a carboxyl group in hemicelluloses, resulting in lignin–carbohydrate covalent linkages (Fig. 4a)^72–74^. In contrast, the quinonemethide intermediate resulting from the analogous 8–O–4 cross-coupling of a hydroxycinnamaldehyde monomer can enter a more facile re-aromatization pathway through elimination of the 8-proton, limiting the nucleophilic attack by hemicelluloses to form lignin–carbohydrate linkages (Fig. 4b)^34,44^. Such reduced lignin–hemicellulose associations may eventually lead to looser lignocellulose assembly in *CAD*-deficient cell walls, thereby resulting in disordered cellulose assembly as observed in our *cad2* rice mutant and also in *Arabidopsis cadc cadd* mutant^12^. It is also notable that our *cad2* rice cell walls display a reduction in cell-wall-bound FA (Fig. 2b), which may also contribute to reducing lignin–hemicellulose associations, because FA can serve as cross-linkers between lignin and hemicelluloses (arabinoxylans) in grass cell walls^9,46,74^.

### Altered cellulose assembly may lead to improved saccharification performance of *CAD*-deficient rice cell walls

Enzymatic saccharification of lignocellulosic biomass can be affected by many factors^69,75^. Nevertheless, the disordered cellulose assembly in our *CAD*-deficient rice cell walls, i.e., increased proportions of mobile amorphous/surface components over rigid crystalline/internal components in cellulose, as gauged by the solid-state NMR and WAXD data in this study (Figs. 5 and 6a), can substantially contribute to improved saccharification performance. In particular, several studies have detected negative correlations between saccharification efficiency and crystallinity indices of biomass^76–79^, albeit with some controversy, especially when specific chemical pretreatments have been applied prior to enzymatic hydrolysis^80–82^. The physical accessibility of cellulose is another important factor affecting biomass saccharification, because intimate contact between cellulolytic enzymes and cellulose substrates is the prerequisite step for enzymatic hydrolysis to occur^69^. As assessed by Simon’s staining assay (Fig. 6b,c), however, we found no significant difference in the relative porosity and accessibility of the cellulose surface between *cad2* mutant and wild-type cell wall samples. Taken together, it is plausible that the improved biomass saccharification performance of our *CAD*-deficient rice can be mainly attributed to increased proportions of cellulose components that are more amenable for enzymatic hydrolysis, rather than changes in porosity and accessibility that affect the physical interactions between cellulose substrates and cellulolytic enzymes. Such altered cellulose assembly may possibly arise from reduced lignin content, modified lignin polymer structures, and/or disrupted lignin-hemicellulose interactions as discussed above.

In conclusion, our results provide a molecular basis for understanding the relationships between altered lignin chemistry, changes in the lignocellulose supramolecular structure, and processability of biomass in *CAD*-deficient mutant and transgenic plants. Future studies may extend the comparative analyses of chemical and supramolecular structures of lignocellulose to other lignin-modifed mutants and transgenic samples in which lignin content and structures are more systematically and variously altered. In particular, we envision that further understanding of the lignin–polysaccharide associations in cell walls and their relationships with lignin chemistry will greatly increase our understanding of the elusive functions of plant cell walls, as well as our ability to manipulate lignocellulose properties for better biomass utilization.

## Methods

### Plant materials

The *cad2* (*gh2*; accession: NE4246) rice mutant used in this study was originally identified from the *Tos17*-insertional mutant population derived from *O. sativa* L. spp. *japonica* cv. Nipponbare^24,83^. The homozygous *cad2* mutant and wild-type lines were grown side-by-side under greenhouse condition^56^. Mature aerial parts were harvested and dried at 27 °C for 30 days. Dried culm straws were then collected by removing the panicle, leaf blade, and leaf sheath. Culm straws were cut into ca. 5 mm pieces with scissors, homogeneously pulverized with a TissueLyser (Qiagen, Hilden, Germany) (3 min at 25 Hz for each precisely weighed 400 mg culm sample), and then extracted with distilled water, methanol, and hexane^84^ to produce CWR samples.

### Cell wall chemical analyses

The Klason lignin assay^85^, thioglycolic acid assay^86^, microscale thioacidolysis^84,87^, and determination of cellulosic (crystalline) and hemicellulosic (amorphous) glycan contents^87^ and alkaline-releasable *p*-hydroxycinnamic acid contents^88^ were conducted as previously described.

### Solution-state NMR

Lignin-enriched cell wall and dioxane-soluble lignin samples for solution-state HSQC NMR were prepared according to the method described previously^51^. Briefly, CWRs (~300 mg) were ball milled using a Fritsch Pulverisette 7 ball-miller (Idar-Oberstein, Germany) with a 45-mL-size ZrO_2_ vessel containing ten ZrO_2_ balls (600 rpm, 12 cycles of 10 min at 5-min intervals) and then digested with crude cellulases (Cellulysin; Calbiochem, La Jolla, CA, USA) as described previously (isolation yields typically ca. 19% and ca. 13% for wild-type and *cad2* mutant CWRs, respectively). Lignin-enriched cell wall samples from three biologically independent plants of wild-type and *cad2* mutant lines were subjected to the NMR analysis. In parallel, dioxane-soluble lignin samples were prepared from pooled culm CWR samples (3 plants/pool). Briefly, culm CWRs (~900 mg) were ball-milled and digested with crude cellulases as described above for lignin-enriched cell wall sample preparations, and then extracted three times with dioxane-water (96:4, v/v, 50 mL) at room temperature for 3 h. The combined supernatant was concentrated to ~1 mL using a rotary evaporator and then precipitated into 200 mL of 0.01 M aqueous HCl. The precipitates were collected by centrifugation (10,000 g at room temperature for 10 min) and washed with distilled water to yield dioxane-soluble lignins (ca. 5% and ca. 2% from wild-type and *cad2* mutant culm CWRs, respectively).

HSQC NMR analysis was performed on a Bruker Biospin Avance III 800US system (800 MHz for ^1^H; Bruker Biospin, Billerica, MA, USA) equipped with a cryogenically cooled 5-mm TCI gradient probe. The solvent was dimethylsulfoxide (DMSO)-*d*_*6*_/pyridine-*d*_*5*_ (4:1, v/v)^49,50^. Adiabatic HSQC NMR experiments were carried out using standard Bruker implementation (“hsqcetgpsp.3”) using acquisition parameters described previously^49,50^. The central DMSO solvent peaks (δ_C_/δ_H_: 39.5/2.49 ppm) were used as an internal reference. Data processing and analysis were carried out as described previously^49–52,87^ using Bruker TopSpin 4.0 (Mac) software (Bruker Biospin, Billerica, MA, USA). Peak assignments were based on comparison with literature data (Tables S3 and S4)^16,35,44,49,50,52,89^. For volume integration of lignin aromatic signals (Table 1, Fig. 3a), we used C2–H2 correlations from **G** and **G′**, C2–H2/C6–H6 correlations from **H**, **S**, **S′** and ***p*CA**, and C2ʹ–H2ʹ/C6ʹ–H6ʹ correlations from **T**. Signals from **H**, **S**, **S′**, ***p*CA** and **T** were logically halved. Volume integrations of lignin inter-monomeric and end-unit linkages (Table 1, Fig. 3b,c), were carried out using Cα–Hα contours from **I**, **II**, **III**, and **III**′, C_9_–H_9_ contours from **I′** and **IV′′**, and C_7_–H_7_ contours from **IV′′**′. Signals from **III** and **III**′ were logically halved.

### Solid-state NMR

Solid-state NMR analysis of rice CWRs was performed on a Bruker Biospin Avance III 800US system (200 MHz for ^13^C; Bruker Biospin, Billerica, MA, USA) with a 4-mm double resonance magic-angle-spinning (MAS) probe and a ZrO_2_ rotor with a KelF-made cap. The ^13^C MAS spectra were measured at 300 K with a MAS frequency of 12k Hz using either ^1^H–^13^C cross-polarization (CP) or ^13^C-directed dipolar decoupling (DD) to create the initial ^13^C magnetization. The CP MAS spectra were collected with a contact time of 2.5 ms and a recycling delay of 4.0 s, whereas the DD MAS spectra were collected using a short recycling delay (2.0 s) to emphasize mobile components. The ^13^C chemical shifts were externally referenced to the CH_3_ signal of silicon rubber at 1.4 ppm on the tetramethylsilane (TMS) scale. Data processing and analysis for CP and DD MAS spectra used Bruker TopSpin 4.0 (Mac) software (Bruker Biospin, Billerica, MA, USA). The ^13^C spin-lattice relaxation time (*T*_1_) measurements were performed using the Torchia pulse sequence^63^ with a CP contact time of 2.5 ms and 12 *τ* delay times between 0.01 and 64 s. The obtained *τ*-dependent signal decay *I*(*τ*) was fitted using Igor Pro 7 software (WaveMetrics, Inc., Portland, OR, USA) with the following double exponential equation:

$$I(\tau )=I{(0)}_{s}{e}^{-\tau /{T}_{1s}}+I{(0)}_{f}{e}^{-\tau /{T}_{1f}}$$

where *T*_1*S*_ and *T*_1*f*_ are two independent *T*_1_ for slower- and faster-relaxing components (*T*_1*f*_ > *T*_1*S*_), and *I*(0)_*s*_ and *I*(0)_*f*_ are their respective weighing fractions^59,64,67^.

### X-ray diffraction analyses

The WAXD analyses were performed on rice CWR powder samples using a Rigaku Ultima-IV diffractometer (Rigaku, Tokyo, Japan) with nickel-filtered CuKα radiation (λ = 1.54 Å) generated at 40 kV and 40 mA. The diffraction intensity profiles of CWR samples placed on a copper sample folder were measured at 20 °C in the 2*θ* range of 5 to 30° with a step size of 0.1° and a scan speed of 15 s per step. The apparent cell wall crystallinity index (CrI) was determined by the height of the 200 peak (*I*_200_, 2*θ* = ca. 22°) and the minimum between the 200 and 100 peaks (*I*_am_, 2*θ* = ca. 18°) using the following equation:

$${\rm{CrI}}( \% )=\frac{({I}_{200}-{I}_{am})}{{I}_{200}}\,\times 100$$

where *I*_200_ is the height of the 200 peak (2*θ* = ca. 22°) and *I*_*am*_ is the minimum between the 200 and 100 peaks (2*θ* = ca. 18°)^65^.

### Simon’s staining assay

The cellulose accessibility of CWRs was determined by the modified Simon’s staining assay using direct orange (DO) and direct blue (DB) dyes according to Chandra *et al*.^68^. Briefly, 20 mg CWR was added to a 15-mL polypropylene centrifuge tube and incubated with increasing concentrations of DO and DB dyes (0.25, 0.5, 0.75, and 1 mg/mL) in phosphate buffer saline solution (pH 6.0). The mixtures were incubated at 70 °C for 6 h with shaking at 150 rpm and then centrifuged. The absorbance of the supernatant was measured using a Shimadzu UV-2600 UV-Vis spectrophotometer (Shimadzu, Kyoto, Japan) at 455 and 624 nm, the peak absorption wavelengths of the DO and DB dyes, respectively. The amount of dye adsorbed by CWR was determined from the difference between the initial dye concentration and the final dye concentration in the supernatant. The concentrations of DB and DO were calculated based on the Lambert–Beer law for binary mixtures and the extinction coefficients for DB and DO dyes at 455 and 624 nm, which were determined by constructing standard curves for each dye^68^.

### Enzymatic saccharification assays

Enzymatic saccharification efficiency was determined as described in Hattori *et al.* (2012)^45^. Briefly, each CWR (~15 mg) sample was placed in a polypropylene tube, destarched, and subjected to enzymatic hydrolysis with an enzyme cocktail composed of Celluclast 1.5 L (Novozymes, Bagsvaerd, Denmark) (1.1 FPU), Novozyme 188 (Novozymes) (2.5 CbU), and Ultraflo L (Novozymes) (65 μg) in sodium citrate buffer (pH 4.8). The reaction was carried out in a rotary reactor (Heatblock Rotator SN-48BN, Nissin, Saitama, Japan) at 12.5 rpm at 50 °C for 24 h. The concentration of liberated glucose was determined with a Glucose CII test kit (Wako Pure Chemicals Industries, Osaka, Japan) according to the manufacturer’s instructions.

## Supplementary information

Supplementary Information

## Data Availability

All data necessary to evaluate the conclusions in this study are included in the published paper and its Supplementary Information file. Additional data, if required, will be made available by the corresponding authors upon request.
