# Supplementary material for: Altered lignocellulose chemical structure and molecular assembly in CINNAMYL ALCOHOL DEHYDROGENASE-deficient rice
Source: Sci Rep. 2019 Nov 20;9:17153. doi: 10.1038/s41598-019-53156-8 (PMC6868246; doi:10.1038/s41598-019-53156-8)
Supplement: Supplementary file 1 — Supplementary Information [file 41598_2019_53156_MOESM1_ESM.pdf]

SUPPLEMENTARY INFORMATION

Altered lignocellulose chemical structure and molecular assembly in *CINNAMYL ALCOHOL DEHYDROGENASE*-deficient rice

Andri Fadillah Martin[1,2,*], Yuki Tobimatsu[1,*,†], Ryosuke Kusumi[3], Naoyuki Matsumoto[1], Takuji Miyamoto[1], Pui Ying Lam[1], Masaomi Yamamura[1], Taichi Koshiba[1], Masahiro Sakamoto[3], Toshiaki Umezawa[1,4,†]

[1]Research Institute for Sustainable Humanosphere, Kyoto University, Uji, Kyoto 611-0011, Japan; [2]Research Center for Biotechnology, Indonesian Institute of Sciences (LIPI), Jl. Bogor KM 46 Cibinong Bogor 16911, Indonesia; [3]Graduate School of Agriculture, Kyoto University, Sakyo-ku, Kyoto 606-8502, Japan; [4]Research Unit for Development of Global Sustainability, Kyoto University, Uji, Kyoto 611-0011, Japan;

[†]Correspondence: Yuki Tobimatsu (ytobimatsu@rish.kyoto-u.ac.jp) and Toshiaki Umezawa (tumezawa@rish.kyoto-u.ac.jp)

List of Materials

Table S1. Growth phenotypes of wild-type and *cad2* rice plants.

Table S2. Saccharification performance of wild-type and *cad2* rice plants.

Table S3. Aromatic peak assignments for HSQC NMR spectra

Table S4. Aliphatic and aldehyde peak assignments for HSQC NMR spectra

Figure S1. Morphological phenotype of wild-type and *cad2* rice plants.

Figure S2. HSQC NMR spectra of lignin-enriched cell wall samples.

Figure S3. WAXD profiles of wild-type and *cad2* rice cell wall powders.

Table S1. Growth phenotypes of wild-type (WT) and *cad2* mutant plants

Trait	WT	*cad2*
Plant Height[a] (cm)	107.1 ± 9.8	123.6 ±5 .2
Culm length[b] (cm)	75.8 ± 5.4	79.9 ± 1.7
Ear length (cm)	19.5 ± 1.8	19.5 ± 1.2
Tiller number	8.0 ± 1.1	9.5 ± 1.0
Ear number	7.8 ± 1.2	10.3 ± 2.2
Dry mass of culm (g)	5.2 ± 1.5	4.9 ± 0.9
Dry mass of leaf sheath (g)	5.4 ± 1.1	5.4 ± 0.8
Dry mass of leaf blade (g)	5.5 ± 0.9	4.8 ± 0.8
CWR yield of culm (%)[c]	48.5 ± 3.2	48.3 ± 3.2

Values are means ± SD (n = 3) [a] Length from cotyledonary node to the tip of the top leaf. [b] Length from cotyledonary node to panicle base. [c] CWR, cell wall residue.

Table S2. Saccharification performance of wild-type (WT) and *cad2* mutant plants

Glucose yield	WT	*cad2*
Per cell walls (mg/g CWR)	180.4 ± 35.4	**238.4 ± 20.2***
Per glucan (mg/g glucan)	346.4 ± 72.3	**471.2 ± 50.5***

Values are glucose yields after 24 h saccharification and means ± standard deviation (SD) from individually analyzed plants (n = 3). Asterisks indicate significant differences between WT and *cad2* mutant plants (Student's *t*-test, *: $p < 0.05$). CWR, cell wall residue.

Table S3. Aromatic peak assignments for solution-state 2D HSQC NMR spectra of rice cell wall and lignin samples

Labels	δ_C/δ_H (ppm)	Assignment	Note
**H**$_{2/6}$	127.9/7.17	C2–H2 and C6–H6 in *p*-hydroxyphenyl units	Potentially overlap with residual proteins [1]
**H**$_{3/5}$	ca. 116/6.8	C3–H3 and C5–H5 in *p*-hydroxyphenyl units	Overlap with **G**$_{5/6}$, ***p*CA**$_{3/5}$ and **G′**$_{5/6}$
**G**$_{2}$	111.2/7.05	C2–H2 in guaiacyl units	
**G**$_{5/6}$	ca.116/6.8,119.3/6.88	C5–H5 and C6–H6 in guaiacyl units	Overlap with **H**$_{3/5}$, **G**$_{5/6}$ and **G′**$_{5/6}$
**S**$_{2/6}$	104.2/6.77	C2–H2 and C6–H6 in syringyl units	
***p*CA**$_{2/6}$	130.3/7.48	C2–H2 and C6–H6 in *p*-coumarate units	
***p*CA**$_{3/5}$	115.6/6.85	C3–H3 and C5–H5 in *p*-coumarate units	Overlap with **H**$_{3/5}$, ***p*CA** $_{3/5}$ and **G′**$_{5/6}$
***p*CA**$_{7}$	145.1/7.63	C7–H7 in *p*-coumarate units	Potentially overlap with residual ferulates
***p*CA**$_{8}$	113.8/6.36	C8–H8 in *p*-coumarate units	Potentially overlap with residual ferulates
**T**$_{3}$	104.9/7.05	C3–H3 in tricin units	
**T**$_{6}$	99.1/6.32	C6–H6 in tricin units	
**T**$_{8}$	94.2/6.60	C8–H8 in tricin units	
**T**$_{2′/6′}$	104.3/7.36	C2′–H2′ and C6′–H6′ in tricin units	
**G′**$_{2}$	111.3/7.33	C2–H2 in cinnamaldehyde-derived guaiacyl units	Potentially overlap with residual ferulates
**G′**$_{5/6}$	ca.116/6.8,123.3/7.12	C5–H5 and C6–H6 in cinnamaldehyde-derived guaiacyl units	Overlap with **H**$_{3/5}$, **G**$_{5/6}$, and ***p*CA**$_{3/5}$
**S′**$_{2/6}$	107.1/7.26, 108.1/7.18	C2–H2 and C6–H6 in cinnamaldehyde-derived syringyl units	
**I′**$_{7}$	138.3/7.41	C7–H7 in cinnamaldehyde-derived 8–O–4 units	
**IV″**$_{7}$	152.9/7.55	C7–H7 in cinnamaldehyde end-units	
**IV″**$_{8}$	127.1/6.81	C8–H8 in cinnamaldehyde end-units	

Measured in DMSO-d_6/Py-d_5 (4:1, v/v). Assignment was based on comparison with literature data: [1] Kim, H. et al. Characterization and elimination of undesirable protein residues in plant cell wall materials for enhancing lignin analysis by solution-state nuclear magnetic resonance spectroscopy. *Biomacromolecules*, **18**, 4184-4195 (2017). [2] Kim, H. & Ralph, J. Solution-state 2D NMR of ball-milled plant cell wall gels in DMSO-d_6/pyridine-d_5. *Org. Biomol. Chem.* **8**, 576-591 (2010). [3] Mansfield, S.D. et al. Whole plant cell wall characterization using solution-state 2D NMR. *Nat. Protoc.* **7**, 1579-1589 (2012). [4] Zhao, Q. et al. Loss of function of cinnamyl alcohol dehydrogenase 1 leads to unconventional lignin and a temperature-sensitive growth defect in *Medicago truncatula*. *Proc. Natl. Acad. Sci. USA* **110**, 13660–13665 (2013). [5] Lan, W. et al. Tricin, a flavonoid monomer in monocot lignification. *Plant Physiol.* **167**, 1284-1295 (2015). [6] Van Acker, R. et al. Different routes for conifer- and sinapaldehyde and higher saccharification upon deficiency in the dehydrogenase CAD1. *Plant Physiol.* **175**, 1018-1039 (2017). [7] Tarmadi, D. et al. NMR studies on lignocellulose deconstructions in the digestive system of the lower termite *Coptotermes formosanus* Shiraki. *Sci. Rep.* **8**, 1290 (2018).

Table S4. Aliphatic and aldehyde peak assignments for solution-state 2D HSQC NMR spectra of rice cell wall and lignin samples

Labels	δ_C/δ_H (ppm)	Assignment	Note
**methoxyl**	3.72/55.7	C–H in aromatic methoxyl groups	
**I$_\alpha$**	71.9/4.99	Cα–Hα in β–O–4 units	
**I$_\beta$**	86.2/4.22	Cβ–Hβ in β–O–4 units	Connected to S aromatic units
	83.5/4.47	Cβ–Hβ in β–O–4 units	Connected to G aromatic units
**I$_\gamma$**	ca. 60.2/3.72	Cγ–Hγ in β–O–4 units	γ-free
	ca. 63.9/4.32	Cγ–Hγ in β–O–4 units	γ-esterifed
**II$_\alpha$**	87.2/5.50	Cα–Hα in β–5 units	
**II$_\beta$**	53.6/3.49	Cβ–Hβ in β–5 units	
**III$_\alpha$**	85.0/4.71	Cα–Hα in resinol-type β–β substructures	
**III$_\beta$**	53.7/3.01	Cβ–Hβ in resinol-type β–β substructures	
**III′$_\alpha$**	82.9/4.99	Cα–Hα in tetrahydrofuran-type β–β substructures	
**III′$_\beta$**	47.5/2.69	Cβ–Hβ in tetrahydrofuran-type β–β substructures	
**IV$_\gamma$**	61.8/4.14	Cγ–Hγ in cinnamyl alcohol end-groups	
**IV′$_\gamma$**	64.3/4.79	Cγ–Hγ in cinnamyl alcohol ester end-groups	
**I′$_9$**	186.2/9.26 (**I′$_{S/9}$**)	C9–H9 in cinnamaldehyde-derived 8–O–4 units	Connected to S aromatic units
	187.7/9.59 (**I′$_{G/9}$**)	C9–H9 in cinnamaldehyde-derived 8–O–4 units	Connected to G aromatic units
**IV″$_9$**	193.8/9.65	C9–H9 in cinnamaldehyde end-units	
**IV‴$_7$**	190.6/9.74	C7-H7 in benzaldehyde end-units	

Measured in DMSO-d_6/Py-d_5 (4:1, v/v). Assignment was based on comparison with literature data: [1] Kim, H. et al. Characterization and elimination of undesirable protein residues in plant cell wall materials for enhancing lignin analysis by solution-state nuclear magnetic resonance spectroscopy. *Biomacromolecules*, **18**, 4184-4195 (2017). [2] Kim, H. & Ralph, J. Solution-state 2D NMR of ball-milled plant cell wall gels in DMSO-d_6/pyridine-d_5. *Org. Biomol. Chem.* **8**, 576-591 (2010). [3] Mansfield, S.D. et al. Whole plant cell wall characterization using solution-state 2D NMR. *Nat. Protoc*. **7**, 1579-1589 (2012). [4] Zhao, Q. et al. Loss of function of cinnamyl alcohol dehydrogenase 1 leads to unconventional lignin and a temperature-sensitive growth defect in *Medicago truncatula*. *Proc. Natl. Acad. Sci. USA* **110**, 13660–13665 (2013). [5] Lan, W. et al. Tricin, a flavonoid monomer in monocot lignification. *Plant Physiol*. **167**, 1284-1295 (2015). [6] Van Acker, R. et al. Different routes for conifer- and sinapaldehyde and higher saccharification upon deficiency in the dehydrogenase CAD1. *Plant Physiol.* **175**, 1018-1039 (2017). [7] Tarmadi, D. et al. NMR studies on lignocellulose deconstructions in the digestive system of the lower termite *Coptotermes formosanus* Shiraki. *Sci. Rep.* **8**, 1290 (2018).

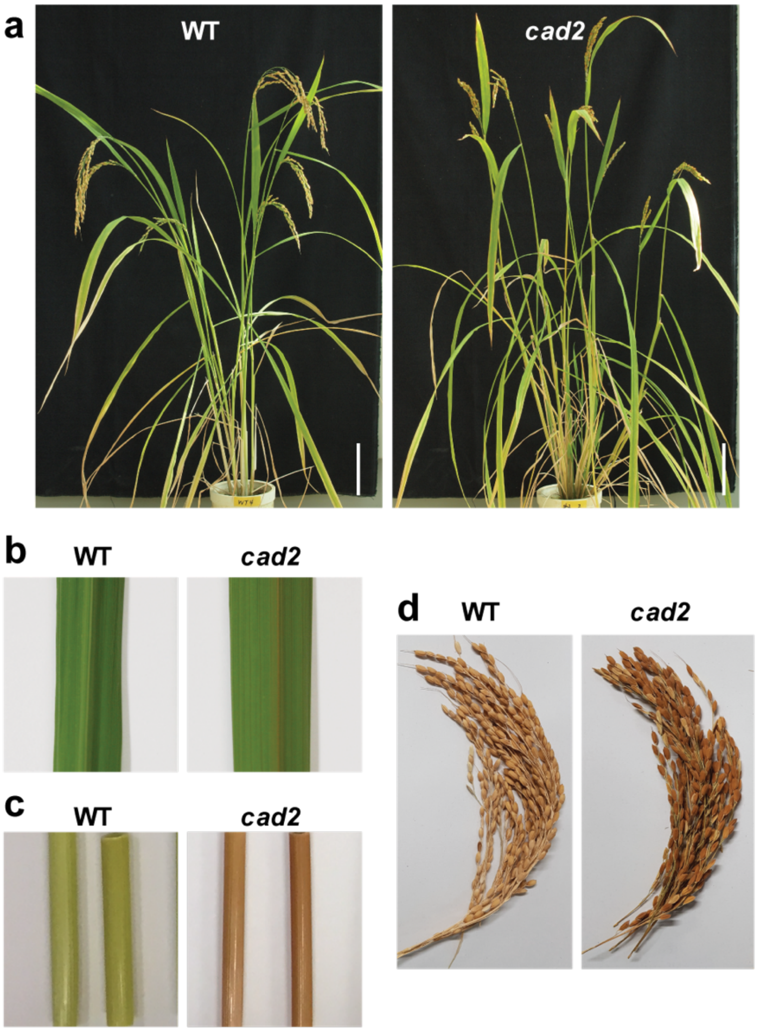

Figure S1. Morphological phenotype of wild-type (WT) and *cad2* mutant rice plants. Plants at the ripening stage (**a**), flag leaves (**b**) and culm straw (**c**) at the heading stage, and harvested panicles (**d**) are shown. Scale bars in **a** denote 10 cm.

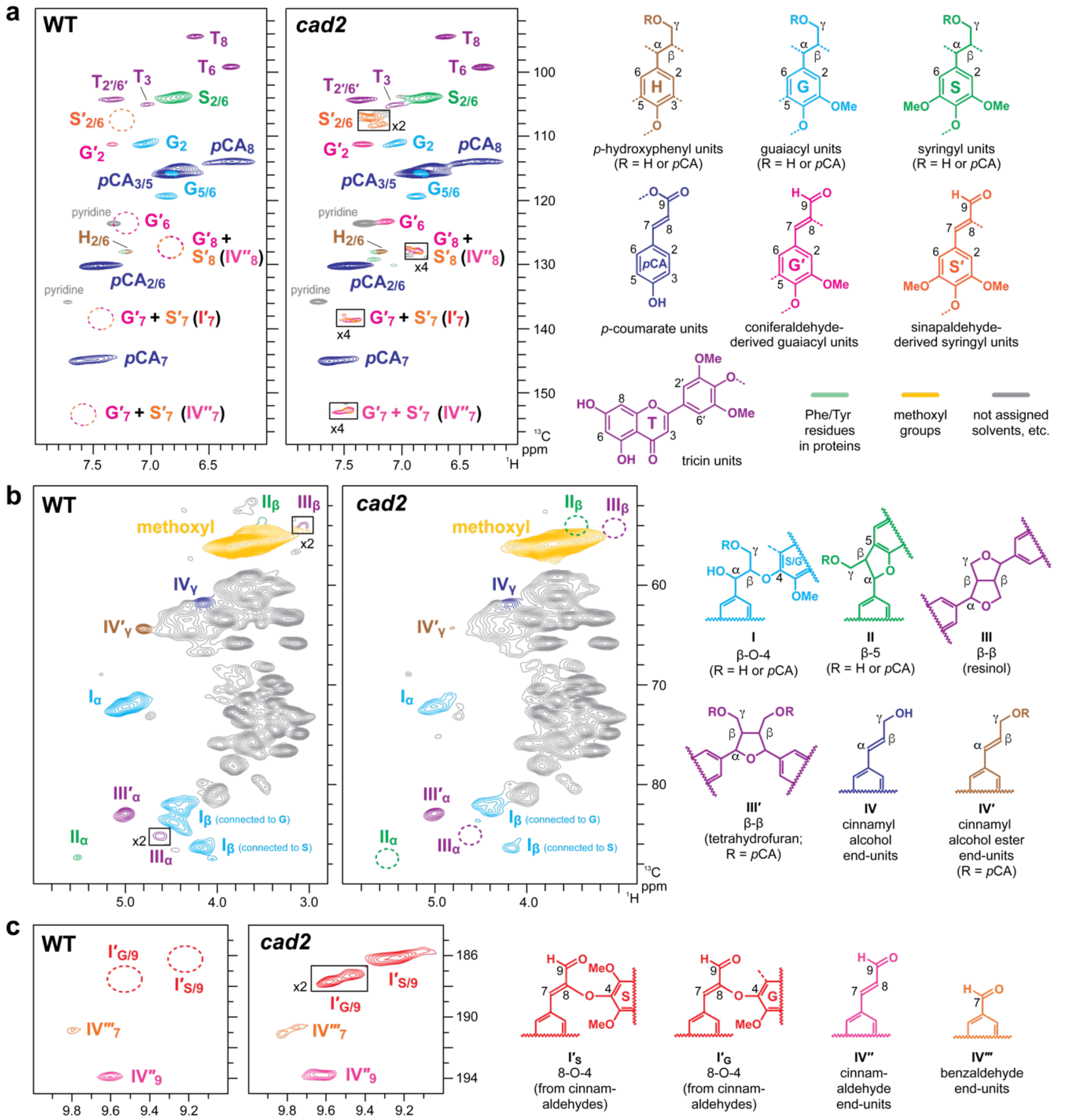

Figure S2. Solution-state two-dimensional short range ^{1}H–^{13}C correlation (HSQC) NMR analysis of lignin-enriched cell wall samples from wild-type (WT) and *cad2* mutant rice plants. (**a**) Aromatic sub-regions showing signals from major lignin aromatic units. (**b**) Oxygenated-aliphatic sub-regions showing signals from major lignin side-chain units. (**c**) Aldehyde sub-regions showing signals from aldehyde units. Contours are color-coded to match with the structures displayed. Boxes labeled ×2 and ×4 represent regions with scale vertically enlarged by 2- and 4-folds, respectively.

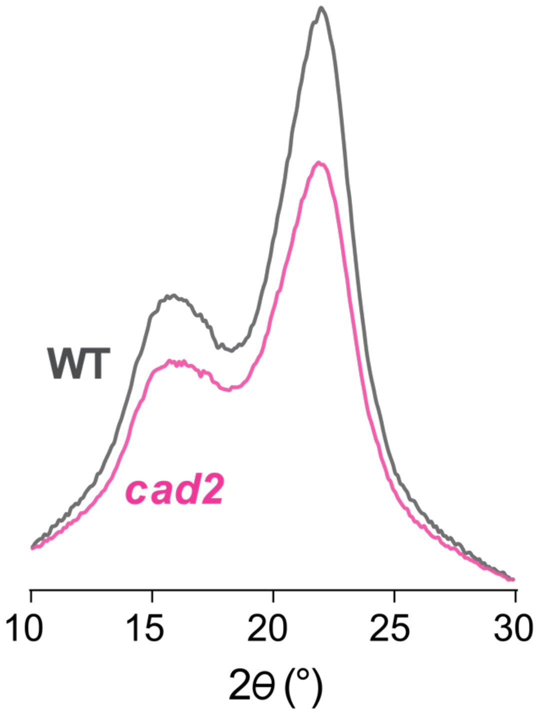

Figure S3. WAXD profiles of wild-type (WT) and *cad2* mutant rice cell wall powder samples.
